# Interdisciplinary Study of the Effects of Dipeptidyl-Peptidase III Cancer Mutations on the KEAP1-NRF2 Signaling Pathway

**DOI:** 10.3390/ijms23041994

**Published:** 2022-02-11

**Authors:** Sara Matić, Ana Tomašić Paić, Sandra Sobočanec, Marija Pinterić, Goran Pipalović, Monika Martinčić, Mihaela Matovina, Sanja Tomić

**Affiliations:** Ruđer Bošković Institute, Bijenička 54, 10000 Zagreb, Croatia; sara.matic@irb.hr (S.M.); ana.tomasic.paic@irb.hr (A.T.P.); sandra.sobocanec@irb.hr (S.S.); marija.pinteric@irb.hr (M.P.); goran.pipalovic@irb.hr (G.P.); monikalo016@gmail.com (M.M.)

**Keywords:** DPP III, KEAP1-NRF2 pathway, oxidative stress, cancer mutation, protein interaction

## Abstract

Dipeptidyl peptidase III (DPP III) is associated with cancer progression via interaction with KEAP1, leading to upregulation of the KEAP1-NRF2 oxidative stress pathway. Numerous DPP III mutations have been found in human tumor genomes, and it is suggested that some of them may alter affinity for KEAP1. One such example is the DPP III-R623W variant, which in our previous study showed much higher affinity for the Kelch domain of KEAP1 than the wild-type protein. In this work, we have investigated the effects of this mutation in cultured cells and the effects of several other DPP III mutations on the stability of KEAP1-DPP III complex using an interdisciplinary approach combining biochemical, biophysical and molecular biology methods with computational studies. We determined the affinity of the DPP III variants for the Kelch domain experimentally and by molecular modeling, as well as the effects of the R623W on the expression of several NRF2-controlled genes. We confirmed that the R623W variant upregulates NQO1 expression at the transcriptional level. This supports the hypothesis from our previous study that the increased affinity of the R623W variant for KEAP1 leads to upregulation of the KEAP1-NRF2 pathway. These results provide a new perspective on the involvement of DPP III in cancer progression and prognosis.

## 1. Introduction

Dipeptidyl peptidase III (DPP III, EC 3.4.14.4) is a zinc metallopeptidase with relatively broad specificity that sequentially cleaves dipeptides from unsubstituted amino termini of 3 to 10 residues long peptides in vitro, preferring substrates with 4 to 8 residues [1,2]. There are several reports on the possible involvement of DPP III in human carcinogenesis. Increased levels and activity of DPP III have been detected in malignant endometrial tissue [3], and expression of DPP III has been positively correlated with ovarian cancer aggressiveness [4].

DPP III is epigenetically induced in liver cancer cells by promoter hypomethylation, while DPP III and thimet oligopeptidase-1 (TOP-1) decrease the immunogenicity of necrotic tumor cells by blocking antigen cross-presentation [5,6]. Higher expression of DPP III correlates with shorter survival of patients with multiple myeloma, and the increased level of DPP III in patients with relapsed multiple myeloma compared to newly diagnosed patients suggests that it may be involved in cancer progression [7].

Apart via its peptidase activity, it is suggested that DPP III may be involved in the development of some tumors via the upregulation of the oxidative stress response pathway KEAP1-NRF2 (Kelch-like ECH associated protein 1—Nuclear factor (erythroid-derived 2]—like 2 protein)) [8,9].

Constitutive repression of NRF2 is achieved by KEAP1, which acts as a dimer such that each monomer binds one motif, either ETGE or DLG, of a single NRF2 protein [10]. NRF2 has two faces, it protects normal cells from the oxidative stress damage, however, it is also often deregulated in cancer where it protects cancer cells from oxidative damage and xenobiotics, including chemotherapeutics. NRF2 also acts by increasing the proliferation of cancer cells through the regulation of a number of genes that control the proliferation of the cells, and some oncogenic proteins increase the transcription of NRF2 [11]. Several mechanisms that increase activity of KEAP1-NRF2 signaling pathway in cancers have been reported. They include somatic mutations in KEAP1, CUL3 or NRF2, epigenetic silencing of KEAP1, accumulation of proteins that interact with KEAP1, transcriptional up-regulation of NRF2, and modification of KEAP1 by metabolic intermediate [12,13]. It is known that proteins with an ETGE/ESGE motif, including DPP III, can act as competitive KEAP1 interactors. Recently, the role of DPP III in oxidative stress was confirmed by the study in DPP III KO mice, where the absence of DPP III was associated with a decrease in NRF2 activity and increased oxidative stress, leading to bone loss through increased osteoclast activity [14], and by the study conducted by Ren at al. [15] on the mouse hippocampal neuronal cell line, HT22, subjected to oxygen-glucose deprivation/reoxygenation (OGD/R). Authors showed that DPP III protects neurons from injury by oxygen-glucose deprivation/reoxygenation via NRF2 signaling.

Our hypothesis was that, in addition to DPP III overexpression, some DPP III mutations found in the human tumor genomes might be involved in the progression of cancer by changing its affinity for KEAP1, and subsequently affecting the KEAP1-NRF2 signaling pathway. However, none of around 260 mutations in the coding regions of DPP III gene listed in cBioPortal cancer genomic database (https://www.cbioportal.org/ (accessed on 17 December 2021)), have been considered as cancer drivers, yet, while 468 of more than 1300 mutants of KEAP1, and 602 of around 1000 mutations in NRF2 listed in this database are regarded as cancer drivers indicating that mutational inactivation might be a common mechanism of the KEAP1-NRF2 signaling disruption in cancer. In the present study we analyzed several DPP III mutant variants whose sequences were found in the cBioPortal database to assess their affinity for the Kelch domain of KEAP1 protein and compare it to that of WT DPP III. Previously, we have shown that the binding of DPP III to Kelch is a two-step process, which involves separation of the ETGE motif, which is located at the tip of the flexible loop in the upper domain of DPP III, from the protein body and insertion of the loop with ETGE motif into the binding site of the Kelch domain. We showed that the DPP III-R623W variant, in which the ETGE loop is less tightly bound to the protein body, has a substantially higher affinity towards the Kelch domain than the WT [16,17]. In this work, we extended our investigations to some other mutations that either shift the equilibrium process of loop detachment or alter the binding affinity of the ETGE loop of DPP III to the Kelch domain.

In order to elucidate whether mutations found in the cBioPortal cancer genomics database affect the binding of DPP III to KEAP1 we combined several experimental and computational approaches.

## 2. Results

**Figure 1:**
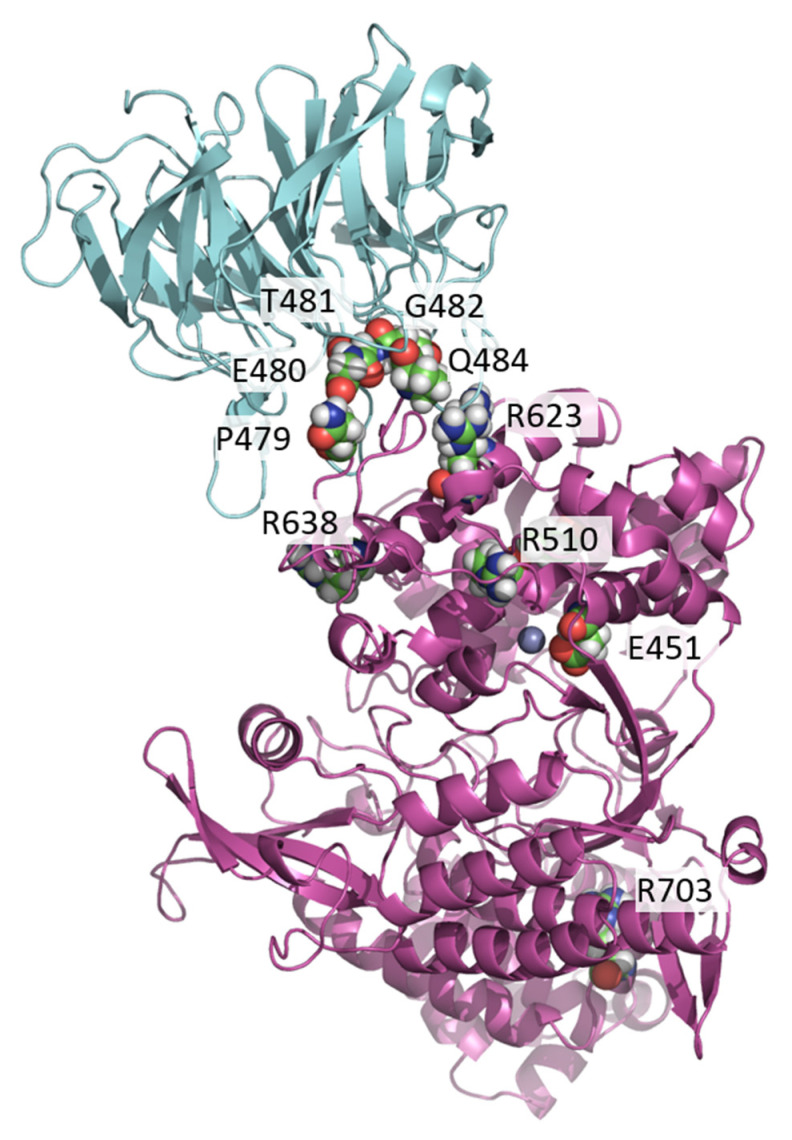
The initial structure of Kelch (cyan)-DPP III (purple) complex with the residues of DPP III selected for mutational study emphasized by spheres representation (colored by element with carbon atoms in green) and labeled. The zinc ion of DPP III is shown as grey sphere.

We selected several mutations in the DPP III gene that have been found in tumor tissues and are listed in cBioPortal cancer genetics database (Figure 1). In an interdisciplinary study, combining several experimental and computational techniques, we investigated their effect on the KEAP1-DPP III affinity.

### 2.1. Peptidase Activity of DPP III Variants

The previously published study identified variants of DPP III that affect its peptidase activity [18]. Therefore, before investigating the relative Kelch-binding affinities of the newly constructed DPP III variants, a kinetic analysis of their peptidase activity was performed using the synthetic substrate Arg_2_-2NA and the kinetic parameters were determined (Table 1).

Most variants tested have a slightly lower *k_cat_* value than the wild-type protein, which is around 70 s^−1^. The lowest *k_cat_* values, almost 10 times lower than that of the wild type, was obtained for the R638W variant, ~8.6 s^−1^, while *k_cat_* of the other mutation affecting the same amino acid, R638L is ~15.6 s^−1^. Interestingly, the lowest *K_M_* values were measured for R638L and R638W (~3.5 μM), also. As a result, their catalytic efficiency is not significantly lower than that of the WT DPP III. Arg638, part of an α helix of the upper domain of DPP III electrostatically interacts with E179 and D186 from the lower domain and in this way boosts the protein globularity and hinder substrate binding. Its mutations to either Trp or Leu improve protein flexibility. As a consequence, substrate can bind to the active site more easily, but the catalytic performances of the protein decreases. With the exception of the E480Q mutation, which did not affect the peptidase activity of DPP III, the other point mutations of the ETGE DPP III loop slightly affected the enzyme activity. This is not surprising considering that the ETGE loop is located in the protein chain between the DPP III motifs HELLGH and EECRAE, which are actively involved in catalysis. It is therefore possible that these substitutions contribute to some extent to changes in the active site of the enzyme and consequently to enzymatic activity. In contrast, mutations in residues R620 and R623 did not alter the peptidase activity of DPP III toward the Arg_2_-2NA substrate.

### 2.2. MST Study of the Effects of DPP III Mutations on its Binding Affinity for the Kelch Domain

Binding of the wild type DPP III (WT) and its variants to the labeled Kelch domain was examined by a binding-induced fluorescence change on the MST instrument, and dissociation constants (*K_d_*) were determined (Table S1. The affinities of the variants compared with the WT DPP III (*K_d_*(WT)/*K_d_*(mut)) is given in Table 2.

Previously, we found that R623W had *K_d_* almost two orders of magnitude lower than WT DPP III (*K_d_* 5 × 10^−9^ mol dm^−3^ and 8 × 10^−7^ mol dm^−3^, respectively). Such a significant increase in affinity for the variant is consistent with our proposed mechanism of binding of DPP III to KEAP1. Namely, DPP III binds to the Kelch domain of KEAP1 via the ETGE motif, which is tightly bound to the protein body by strong hydrogen bonds with R623. Mutation of R623 to Trp facilitates the release of the ETGE motif and its binding to Kelch.

In this work, we considered the mutations of DPP III listed in cBioPortal, that are located in its upper domain, either in the ETGE motif, in the loop to which the ETGE motif belongs, or in the regions that we identified in our previous MD studies as interacting with the ETGE motif. We also considered the mutation of R703, the amino acid located in the lower domain of DPP III, far from the ETGE loop and active site of the enzyme (Table 2).

As expected, mutation of the amino acid residues in the ETGE motif, E480Q, T481M, and G482C, decreased the affinity of DPP III for Kelch, whereas mutations of the residues immediately upstream and downstream of this motif, P479S and Q484H, increased the affinity of DPP III for Kelch (Table 2). The measured binding of the DPP III E480Q and T481M variants to Kelch was an order of magnitude weaker than that of the WT protein. Compared with the result for the DPP III variant with a deletion of the ETGE motif, this would suggest that binding of these variants is present but significantly weaker, consistent with the results of native PAGE and DLS (data not shown) and predictions based on structural analysis. Mutation of Pro479, the residue at the position immediately upstream of the ETGE motif, to Ser, P479S, resulted in a decrease in *K_d_* of about one order of magnitude. This is not surprising since NRF2 protein, the main interactor of KEAP1 has Glu in this position.

Other mutations examined showed no significant effect on altering the binding affinity of DPP III to the Kelch domain of KEAP1. Among them, R510W had the lowest affinity, about one-third that of the wild-type protein, while the affinity of the inactive variant E451K, the variants with substitution of R638 (L and W), and the variant with the mutation at the position at the lower domain R703C showed a slightly higher affinity than that of the wild-type DPP III.

### 2.3. MD Simulations and Computational Analysis

**Figure 2:**
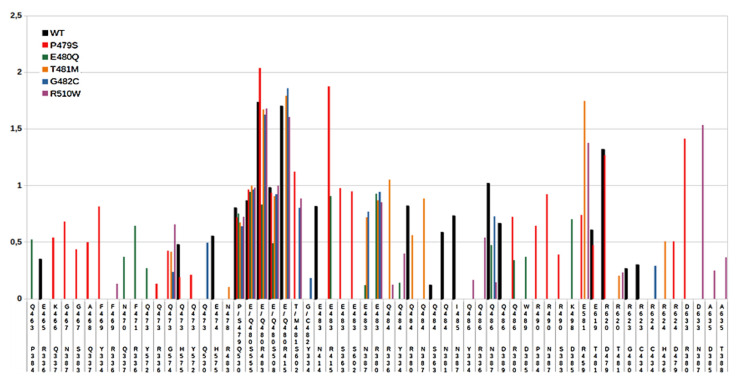
Distribution of the intermolecular hydrogen bonds determined in the 20 ns long interval where the lowest MM-GBSA energy was calculated.

To understand the effects of the point mutations of DPP III on the binding affinity to Kelch at the structural level, we performed MD simulations of the complexes with the variants whose affinity is significantly different from the affinity of the WT proteins. The variants with the point mutation in the ETGE motif, E480Q, T481M, G482C, and immediately upstream of the ETGE motif, P479S, as well as the mutation near the active site, R510W, were selected for MD simulations of the DPP III complex with the Kelch domain. Three independent MD simulations, two with a duration of 200 ns and one with a duration of 300 ns, were performed for WT DPP III and the selected variants, and the stability of the complexes was evaluated by analyzing the structural parameters and the population of hydrogen bonds. In addition, the MMGBSA energies were calculated in the 20-ns intervals of the trajectories. The relative affinity of the protein complexes estimated by the MM-GBSA energy calculations (Table 3) shows greater stability of the complexes with the wild-type protein compared to the complexes with other DPP III variants with mutation in the ETGE motif as well as the complex with the R510W variant, while the least favorable binding was determined for E480Q. A significant decrease in intermolecular interactions was observed in the simulations of the complex Kelch-DPP III E480Q, as shown by the population of intermolecular hydrogen bonds throughout the simulation time and in the interval where the lowest MM-GBSA energy was calculated (Table 4 and Figure 2). The MM-GBSA energy determined for the R623W variant is similar to that of the R510W variant. However, as mentioned above, the high binding affinity determined for the R623W variant is primarily due to the significantly lower work required to detach the ETGE loop from the protein body (Figure S1). Detachment of the ETGE motif from the protein body to which it is bound via strong intramolecular interactions with Arg623 (and Arg624) is the first step in DPP III-Kelch complexation. The R623W mutation significantly weakens these interactions, allowing the ETGE loop to more easily detach from the structural part of DPP III and bind to the Kelch domain of the KEAP1 protein.

**Figure 3:**
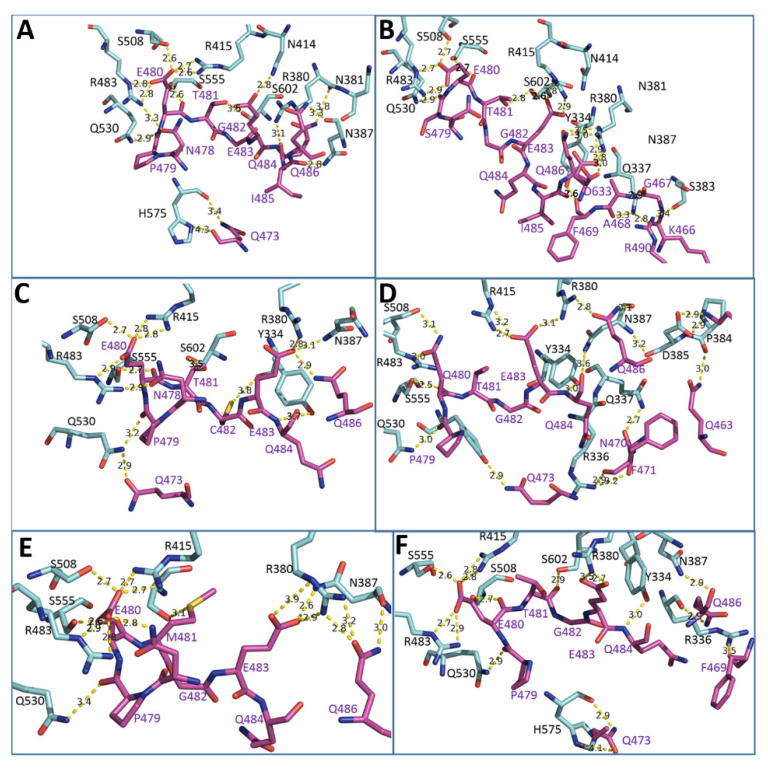
Interactions between the ETGE DPP III motif and the Kelch domain determined during MD simulations of the complex Kelch-DPP III, with DPP III variants (**A**) WT (**B**) P479S, (**C**) G482C, (**D**) E480Q, (**E**) T481M and (**F**) R510W. Residues involved in intermolecular hydrogen bonding are shown as sticks, DPP III residues are colored purple and residues of the Kelch domain colored cyan. The distances between the atom pairs forming hydrogen bonds are marked.

Mutation of P479 to Ser showed a significant effect in terms of the number and pattern of interactions of DPP III with the Kelch domain compared with the wild-type protein (Table S2 and Figure 2 and Figure 3). Strikingly, residue R415 reoriented from E480 to E483, the second glutamate in the ETGE motif, in this variant, and the interactions of residues T481 and E483 with S602 and S363 increased compared with WT. Similar interactions are present in the NRF2-Kelch complex [19]. By mutating P479 to Ser the interactions between DPP III and Kelch became more similar to NRF2 peptide-Kelch interactions. This is not surprising since mutation of P479 to Ser makes the KEAP1-interacting loop of DPP III more similar to the KEAP1-interacting loop of NRF2, the main interactor of KEAP1 [19]. Interestingly, in addition to the interactions between the ETGE loop and Kelch, the P479S-Kelch complex is characterized by strong interactions between the turns connecting the β-strands of the Kelch domain, in particular residues D479, G480, T481, N482, C434, and H436, and the positively charged region at the surface of the highly structured part of DPP III, R620, R623, and R624, which interacts with the ETGE motif in the free form DPP III. Further strong stabilization of the Kelch-DPP III complex is achieved by the interactions between R490 and the Kelch residues on the opposite side of the central pore, N387, S383, P384 and D385.

Note that NRF2 has an EETGE sequence instead of a PETGE sequence in DPP III. Thus, the P479S mutation increases the similarity between DPP III and NRF2, the strongest interactor of KEAP1.

The most pronounced reduction in protein-protein interactions was observed in the case of the E480Q mutation of DPP III, where the loss of interaction of Q480 with R415 leads to a reorientation of R415 toward E483, which also interacts with the Kelch residue R380 (Figure 2 and Figure 3). However, this interaction is not as stable as in the P479S variant. Moreover, Q483 achieved negligible interactions with the Kelch domain during simulation compared to WT. Thus, the interaction of this residue with serine residues S555 and S508 of the Kelch domain is quite stable during the simulations, while R483 of the Kelch domain moved away from the ETGE motif. This is consistent with the worst binding energy obtained by the MM-GBSA calculations for the complex with this DPP III variant.

The T481M substitution affected the intermolecular interactions between Kelch and DPP III (Figure 2 and Figure 3) less than E480Q, which was expected since recent studies have shown that proteins with an ESGE motif can also bind to the Kelch domain of KEAP1 [8]. Although the transient interaction between T481 and S602 of the Kelch domain found in the complex with WT DPP III during the MD-simulations is absent in the complex with the T481M variant, there is an increase in the interactions of E483 with R380 and N387 and of Q484 with R336 and N387 that partially compensate for the absence of interactions due to substitution and the resulting total number of hydrogen bonds, which is only slightly lower than in the WT protein complex. This is consistent with the MM-GBSA energy calculations for this variant compared to WT.

As expected from the lower binding affinity of the G482C variant to Kelch predicted with the MM-GBSA calculation, the G482C mutation shows a decrease in the number of interactions of the ETGE loop with the Kelch domain binding site (Figure 2 and Figure 3). While C482 shows a weak interaction with Y334 and some interactions occur between residue E483 of the ETGE motif and Kelch residues R380 and N387, there is a significant change in the loop conformation that weakens other interactions in the WT protein complex, mainly with residues E483, Q484, and Q486.

### 2.4. Overexpression of DPP III Induces the Expression of NQO1

**Figure 4:**
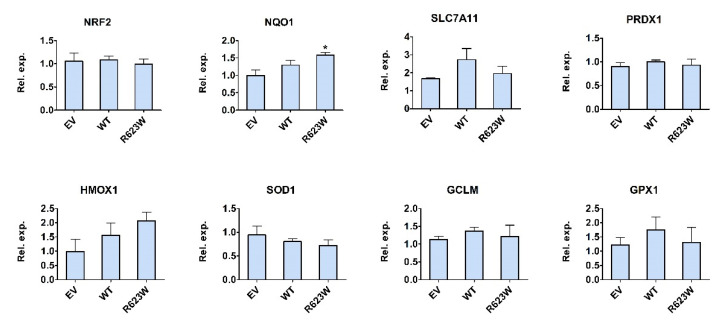
Relative expression of NRF2, NQO1, SLC7A11, PRDX1, HMOX1, SOD1, GCLM, and GPX1 mRNA in HEK293T cells transfected with empty pFLAG-CMV2 vector (EV), WT DPP III and DPP III-R623W variant, respectively, and treated with H_2_O_2_. The results represent the average of 3 biological replicates with standard error. Statistical analysis was performed using one-way ANOVA with Dunnet’s post-hoc test (NQO1: * *p* < 0.05, R623W vs. EV).

HEK293T cells grown in 6-well plates were transfected with the pFLAG-CMV2 empty vector (EV) and vectors for the expression of WT or the R623W variant of the DPP III protein, respectively, and then treated with 400 µM H_2_O_2_. The RNA was isolated and reverse transcribed and the resulting cDNA was analyzed by RT-qPCR to determine whether the expression of WT or the DPP III variant affects the expression of 8 NRF2 controlled genes, NRF2, NQO1, HMOX1, PRDX1, SOD1, GCLM, SLC7A11 and GPX1. The results of the expression analysis are shown in Figure 4. The only significant difference was in the expression of NQO1 between cells overexpressing DPP III-R623W and the EV-transfected cells. The differences in the expression of other NRF2-controlled genes between DPP III overexpressing cells and EV-transfected cells were not statistically significant.

**Figure 5:**
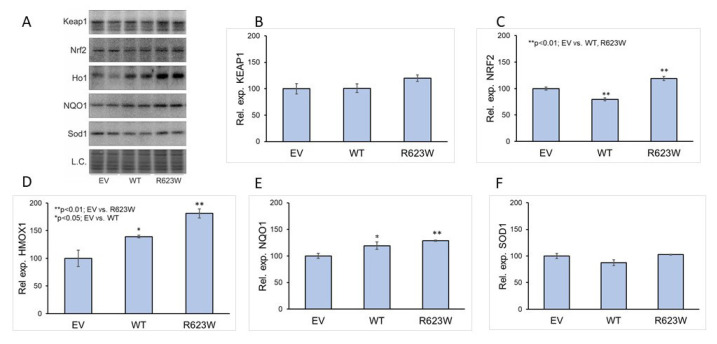
Western blot analysis of the relative expression of KEAP1, NRF2, HMOX1 (Ho1), NQO1 and SOD1 in the cells transfected with EV, WT and R623W, respectively. (**A**), A graphical display of the averaged densitometry values obtained by the analysis of the western blot bends in ImageLab v. 6.1.0. (Bio-Rad Laboratories, Inc., Hercules, CA, USA), normalized to loading control (L.C.) KEAP1 (**B**), NRF2 (**C**), HMOX1 (**E**), NQO1 (**D**) and SOD1 (**F**) protein expression is presented. For the statistical analysis of data, SPSS for Windows (v.17.0, IBM, Armonk, NY, USA) was used. (NRF2: ** *p* < 0.01 EV vs. WT, ** *p* < 0.01 EV vs. R623W; HO1: * *p* < 0.01 EV vs. R623W, ** *p* < 0.05 EV vs. WT; NQO1: * *p* < 0.01 EV vs. R623W, ** *p* < 0.05 EV vs. WT).

In the first biological replicate of the western blot analysis, we found significant upregulation (*p* < 0.01) of NRF2, NQO1 and HMOX1 (Ho1) in cells transfected with the R623W variant, and significant (*p* < 0.05) upregulation of NQO1 and HMOX1 in cells transfected with WT DPP III compared to the EV-transfected cells, whereas NRF2 was significantly (*p* < 0.01) downregulated in WT transfected cells (Figure 5). However, the results obtained after performing three more biological replicates showed no difference in protein expression between cells transfected with EV and DPP III, WT and R623W (Figure S3).

## 3. Discussion

Several studies suggest that DPP III may be involved in the cancer progression. However, the mechanisms of its possible involvement in carcinogenesis are still unclear. One possibility for the involvement of DPP III in cancer progression is its interaction with KEAP1 and the resulting upregulation of the KEAP1-NRF2 pathway, as it is known that the KEAP1-NRF2 pathway is frequently upregulated in cancer. There are several mechanisms for the activation of the KEAP1-NRF2 pathway in cancer, including somatic mutations in KEAP1 or NRF2 that disrupt their binding, or accumulation of interfering proteins [20]. Both KEAP1 and NRF2 are considered as cancer drivers in multiple cancer types, however, most of the mutations found are still lacking experimental conformation [21]. We have previously shown that some DPP III variants have a higher affinity for KEAP1 that could lead to the upregulation of KEAP1-NRF2 signaling pathway. In this work, we analyzed the influence of several DPP III mutations found in the cancer samples on the interaction with the Kelch domain of KEAP1 protein.

Currently, 262 unique mutations in the coding regions of the DPP III gene are listed in the curated set of non-redundant studies on the cBioPortal for Cancer Genomics (https://www.cbioportal.org/ (accessed on 17 December 2021)). We selected 8 mutations in the regions of DPP III important for binding to KEAP1, two in the active site of the enzyme and one in the region distant from the two previously mentioned, purified the variant proteins, and determined the effects of the mutations on the affinity of DPP III for the Kelch domain and on the enzymatic activity. In addition, five of the variant proteins that we considered particularly relevant due to their position in the ETGE motif or which showed significant change in either binding affinity for Kelch or peptidase activity were analyzed by molecular modelling. All mutant variants had lower turnover number and the catalytic efficiency than the WT, so we can exclude the possibility that the potential role of these mutations in cancer development could by assigned to the increased activity of DPP III. MST measurements showed that mutation of amino acid residues in the ETGE motif, E480Q, T481M, significantly decreased the affinity of DPP III for Kelch, while mutation G482C had no effect on the interaction between Kelch and DPP III. Lu et al., 2017 [9] also investigated how mutations in the ETGE motif affect the binding of DPP III to KEAP1. They mutated T481 and G482 to Glu and found that these mutations significantly decreased the affinity of DPP III for KEAP1. This result is consistent with the two-step mechanism of binding of DPP III to KEAP1 that we proposed [17]. Namely, the Glu at positions 481 and 482 could form strong hydrogen bonds with the arginines (R620, R623, and R624) at the top of the structural part of the upper DPP III domain, preventing the detachment of the ETGE motif from the protein surface and its interaction with the Kelch domain of the KEAP1 protein. Similarly, the T481M substitution studied in our work showed a significant decrease in the affinity in MST measurements. In contrast, the G482C mutation found in cBioPortal and also examined in this study was more conservative and did not have such pronounced effect on binding.

In contrast to the mutations in the ETGE motif, mutation of the residue adjacent to the ETGE motif, P479S, increased the affinity of DPP III for Kelch by about an order of magnitude (Table 2). The latter is consistent with the results of Cino et al. 2013 [22], who studied the binding of various peptides of proteins that interact with the Kelch domain. They found that not only the amino acid residues at the position of the ETGE motif itself, but also the amino acid at the position upstream of this motif significantly affected the binding affinity of peptides to Kelch, with Glu, the amino acid present in NRF2, having the greatest benefit. Apparently, the replacement of Pro by Ser makes the polarity of this site more similar to the NRF2 motif. The obtained experimental results are in agreement with our hydrogen bonding analysis and MM-GBSA calculations. On the other hand, R623W binds about two orders of magnitude stronger to the Kelch domain than the WT protein, which is consistent with the proposed mechanism of loop detachment as a crucial step for DPP III-Kelch complexation. Investigation of DPP III KO mice have confirmed its role in the oxidative stress response. The lack of DPP III was related to the decrease in NRF2 activity, and increased oxidative stress that caused bone loss due to the increased activity of the osteoclasts [14], while another study found that the male DPP III KO mice have the enhanced production of ROS in the kidneys [23]. Recently, it was also shown that DPP III suppresses neuronal apoptosis, oxidative stress and inflammation in the oxygen-glucose deprivation/reoxygenation-injured hippocampal neurons through the modulation of KEAP1-NRF2 signaling [15] It has been shown previously that H_2_O_2_ treatment induces the interaction of endogenous DPP III and KEAP1 proteins and increases the expression of NRF2-controlled gene NQO1 [9,24]. Hast et al. showed that silencing of the DPP3 gene by siRNA reduced the expression of the NRF2 target genes HMOX1 and GCSm to a similar extent as silencing NRF2 itself [8]. Silencing of another KEAP1 interactor, FAM129B, has also been shown to reduce NQO1 levels [25]. We investigated whether overexpression of the DPP III mutant R623W in combination with H_2_O_2_ treatment affects the expression of several NRF2-controlled genes at both the transcriptional and translational levels. The R623W variant was selected because it has a much higher affinity for the Kelch domain of KEAP1 than the WT. Both, WT DPP III and the R623W variant were overexpressed in HEK293T cells to determine their effect on the expression of various NRF2-controlled genes after treatment with H_2_O_2_ in comparison to the expression of the same genes in the EV-transfected cells that served as controls. It was found that the R623W variant significantly upregulated the expression of NQO1 mRNA compared to EV-transfected cells. This supports the hypothesis from our previous study that the increased affinity of the R623W variant for KEAP1 protein may be involved in cancer progression through upregulation of the KEAP1-NRF2 pathway. We also detected significant changes in the protein levels of NQO1 and HMOX1 in both, WT DPP III and R623W transfected cells, however, we did not find any difference in the protein expression in the repeated experiments (Figure S3). The discrepancies between biological replicates were substantial and the effect of overexpression of DPP III on the expression of NRF2-controlled gene mRNA was not as strong as we expected, but this may be due to the fact that HEK293T cells endogenously express DPP III. Therefore, we are planning to perform future studies of the effects of DPP III mutations on the KEAP1-NRF2 signaling pathway in the DPP III KO cells.

## 4. Materials and Methods

### 4.1. Protein Expression and Purification

The WT DPP III was cloned into the expression vector pLATE31, and the plasmid pET15b with Kelch insert for the expression of the Kelch domain of KEAP1 protein with N-terminal His-tag was a kind gift from prof. Mark Hannink from the University of Missouri, Columbia, MO, USA. Mutations in DPP III protein were introduced using the QuickChange Site-Directed Mutagenesis kit (Agilent Technologies, Santa Clara, CA, USA), with primers containing target mutations (Table S3. Wild-type proteins DPP III and Kelch, as well as the DPP III variants, containing the His-tag at their C and N terminus, respectively, were expressed in *E. coli* BL21–(DE3)–RIL + strain and purified by affinity chromatography on Ni-NTA agarose [26]. Protein concentrations were determined using BioDrop for measuring protein A280 adjusted by their mass extinction coefficient.

### 4.2. Enzyme Kinetic

The kinetic parameters for the hydrolysis of the synthetic substrate Arg_2_-2NA by WT DPP III as well as variants P479S, E480Q, T481M, G482C, Q484H, R620C, R623W, R623L, R638L and R638W were determined at 25 °C and pH 8.6, in the presence of 50 μM CoCl_2_, by initial rate measurements, as previously described [27]. The parameters of Michaelis-Menten kinetics were calculated by non-linear regression analysis in GraphPad Prism 5.0.1 for Windows (GraphPad Prism 5.0.1, San Diego, CA, USA).

### 4.3. Binding Affinity

For MST measurements, the Kelch domain was fluorescently labeled with RED-MALEIMIDE (Cysteine reactive) fluorescent dye (NanoTemper, München, Germany), and Nano-RED excitation (20%) was used. Measurements were carried out on Monolith NT.115 (NanoTemper, München, Germany) at 25 °C in 50 mM Tris, 100 mM NaCl, pH 8.2, 0.05% Tween buffer, in Monolith NT.115 capillaries. The concentration of the labeled Kelch protein was 50 nM.

### 4.4. Computational Study

#### 4.4.1. System Preparations, Parameterization and Simulations

The structure of the complex used in this study was the one obtained in our previous work. Arginine and lysine residues in our models were positively charged, while glutamate and aspartate residues were negatively charged, as expected under physiological conditions. The protonation states of histidines were determined manually based on their potential to form hydrogen bonds with the neighboring amino acid residues and to coordinate the Zn^2+^ ion within the active site. All systems were parameterized using the ff14SB force field [28]. The zinc ion within the active site was described using the recently developed hybrid bonded/non-bonded model for the metallopeptidases [29]. The system was solvated using a truncated octahedron of TIP3P water molecules [30] and Na^+^ (and/or Cl^−^) ions [31] were added to achieve electroneutrality or desired salt concentration. All MD simulations were carried out using the AMBER16 program suite [32].

#### 4.4.2. Classical MD Simulations

Prior to the productive MD simulations, the systems were optimized in three cycles with different restraints. The first cycle (1500 minimization steps) aimed to relax the solvent molecules, while the protein and the zinc ion were constrained using a harmonic potential with a force constant of 32 kcal mol^−1^ Å^−1^. In the second cycle (2500 minimization steps) only the protein backbone was constrained using the force constant of 12 kcal mol^−1^ Å^−1^ while the whole system was minimized with no additional restraints in the third cycle (1500 minimization steps). The systems were heated from 0 to 300 K during 100 ps long MD simulations followed by 1 ns long equilibration at 300 K. A time step of 1 fs was used for the heating and equilibration simulations. In the productive MD simulations, we used the SHAKE algorithm [33,34] and a 2 fs time step. During heating, the NVT ensemble was utilized, while equilibration and production MDs were performed with the NPT ensemble, with the cutoff value of 11 Å. In all simulations, the pressure was regulated with the Berendsen barostat [35] with the relaxation time of 1.0 ps. Temperature was controlled using the Langevin thermostat [36] with a collision frequency of 1 ps^−1^. A total of 700 ns (2 independent simulations of 200 ns and one of 300 ns) of productive classical MD simulations was performed for each constructed Kelch-DPP III complex.

#### 4.4.3. Data Analysis

Distance calculations, hydrogen bond analysis and other geometric parameters were calculated using the module cpptraj of the AmberTools16 program package [37]. Default cut-off values of 3.0 Å and 135° were used in definition of hydrogen bonds. The hydrogen bond population is given as the ratio of frames containing the bond and the total number of frames sampled during simulations. MMPBSA energies were calculated at the 20 ns intervals throughout the trajectory using the MMPBSA.py script as implemented in AMBER16 suite of programs [38]. An internal dielectric constant of 2 was used, since it has given good results in our previous works [39,40], while the ionic strength was set to 0.100 mol dm^−3^. Figures were prepared by PyMOL (PyMOL Molecular Graphics System, version 1.5.0.4, Schrödinger LLC, New York, NY, USA).

### 4.5. Cloning and Cell Culture

WT DPP3 cDNA was cloned in the pFLAG-CMV2 vector with In-Fusion cloning kit (Takara Bio, Kusatsu, Shiga, Japan) for the expression of FLAG-DPP III in mammalian cell lines. Expression plasmids for mutated variant, FLAG-DPP III-R623W was prepared with QuikChange XL Site-Directed Mutagenesis Kit (Agilent).

Human embryonic kidney 293T (HEK293T) cells were grown in complete high glucose (4.5 g/L) Dulbecco’s Modified Eagle’s medium (DMEM; Sigma Aldrich, Saint Louis, MO, USA) with the addition of 10% fetal bovine serum (FBS), 1% non-essential amino acids and 1% antibiotic/antimycotic solution (all purchased by Capricorn Scientific GmbH, Ebsdorfergrund, Germany). Human cells were, prior to qPCR experiments, examined for mycoplasma contamination according to Sung et al. [41]. No mycoplasma infection was detected.

HEK293T adherent cells were grown in 6-well TPP plates to 70–80% confluency and then transfected with empty pFLAG-CMV2 vector (EV), and pFLAG-CMV2 with WT-DPP3 and mutated variant DPP3-R623W, respectively, with the use of Lipofectamine 2000 (Invitrogen, Waltham, MA, USA) according to the manufacturer’s protocol. Cells were grown for 24 h after transfection and then treated with 400 µM H_2_O_2_ (Sigma) for another 24 h, after which they were harvested for RNA or protein isolation.

### 4.6. RNA Isolation and RT-qPCR

RNA was isolated with Trizol reagent (Ambion, Austin, TX, USA) from the cultured HEK293T cells according to the manufactures protocol. The concentration of RNA was measured on BioDrop (Biochrom, Cambridge, UK) and 1 µg of RNA was used for cDNA synthesis with High Capacity RNA-to-cDNA kit (Applied Biosystems, Waltham, MA, USA). RT-qPCR was performed on ABI 7300 Applied Biosystem (Waltham, MA, USA) to determine the relative level of expression of 8 NRF2-regulated genes, NRF2, NQO1, HMOX1 PRDX1, SOD1, GCLM, SLC7A11 and GPX1 with the use of Power SYBR Green PCR Master Mix (Applied Biosystems, Waltham, MA, USA) with gene-specific primers listed in the Table S4. The expressions of NRF2, NQO1, HMOX1, PRDX1 and SLC7A11 were normalized against the expression of GAPDH as the reference gene, while the expressions of SOD1, GCLM and GPX1 were normalized against the expression of TUBG1 as the reference gene. Melting curve analysis of the amplification reactions confirmed that single amplicon was amplified in each reaction. Amplicons were also analyzed by agarose gel electrophoresis, which confirmed that the amplicons were of the right size. PCR efficiencies of each reaction were determined by the amplification of the serial dilutions of the cDNA sample from untreated cells. PCR efficiencies are listed in Table S4. Reference genes were selected based on the similarity between reaction efficiencies. Relative expression in the cells treated with 400 µM H_2_O_2_ was compared to the expression in the untreated control transfected with the EV. The analysis was performed in three biological replicates, and the results were analyzed by Pfaffl method for the relative quantification of RT-PCR data, which takes into account reaction efficiencies of the amplification for each gene [42]. Statistical analysis was performed using SPSS for Windows (v.17.0, IBM, Armonk, NY, USA). To determine the differences between the treatment groups and the control group (EV), a one-way analysis ANOVA followed by Dunnett’s post hoc test were performed. 

### 4.7. RNA Isolation Protein Isolation and Western Blot

Total cellular proteins for Western blot analysis were isolated in Ripa buffer (50 mM Tris buffer, 150 mM NaCl, 0.1% SDS, 12 mM Na-deoxycholate, 1% Triton) with protease inhibitors (Roche, Basel, Switzerland). Protein concentration was determined by Pierce BCA Protein Assay Kit (Thermo Fischer Scientific, Waltham, MA, USA). Proteins (15 µg/µL) were resolved by SDS-PAGE and transferred onto a PVDF membrane (Roche, Basel, Switzerland). Membranes were blocked and incubated with primary antibodies overnight at 4 °C. For chemiluminescence detection, an appropriate horseradish peroxidase (HRP)-conjugated secondary antibody was used. AmidoBlack (Sigma Aldrich, Saint Louis, MO, USA) was used for total protein normalization. The Alliance Q9 Mini imaging system (UVITEC, Cambridge, UK) was used for the detection of immunoblots using an enhanced chemiluminescence kit (Thermo Fischer Scientific, Waltham, MA, USA). Primary antibodies used in the study: Anti-Heme oxygenase antibody (Abcam, Cat.No: ab13243, Cambridge, UK), Anti-Superoxide Dismutase 1 (Abcam, Cat.No: ab16831, Cambridge, UK), Anti-Nrf2 antibody (Abcam; Cat.No: ab62352, Cambridge, UK), Anti-Nqo1 antibody; (Abcam, Cat.No: ab80588, Cambridge, UK), Anti-Keap1 antibody (Proteintech, Cat.No: 60027-1-Ig, Rosemont, IL, USA).

Secondary antibodies: Anti-rabbit IgG, (H+L) Peroxidase Conjugated (ThermoScientific; Cat.No: 31460, Waltham, MA, USA), Anti-mouse IgG, (H+L) HRP Conjugated; Bio-Rad (Cat.No: 170-6516, Hercules, CA, USA).

## 5. Conclusions

In this work, we investigated how some of the DPP III mutations listed in the cBioPortal cancer genome database affect enzyme activity, the affinity of DPP III for the Kelch domain of the KEAP1 protein, and the KEAP1-NRF2 pathway. We have shown that the R623W and Pro479 mutations positively affect the affinity of DPP III-KEAP1 and R623W increases the expression of some NRF2 controlled genes such as NQO1 (upregulation of the KEAP1-NRF2 pathway of the oxidative stress response). We have also shown that these two mutations affect different stages of DPP III binding to KEAP1. While the R623W mutation facilitates the detachment of the loop containing the ETGE motif, which is critical for the binding of DPP III to KEAP1, from the structured part of the upper DPP domain, the P479S mutation increases the strength of the interaction between the DPP III loop and the Kelch domain of KEAP1. This is not surprising since mutation of Pro479, the residue at the position immediately upstream of the ETGE motif, to Ser makes the polarity of this part of the loop DPP III more similar to the EETGE motif in the NRF2 protein, which has the highest known affinity for KEAP1. Our results suggest that it is important to study the mutations of DPP III found in cancer to gain more insight into the mechanism of their possible involvement in cancer progression.

## Figures and Tables

**Table 1:** Kinetic parameters for Michaelis-Menten kinetics determined by nonlinear regression analysis of data in GraphPad Prism 5.0.1 (results shown represent the mean of 3 measurements with SD).

	WT	P479S	E480Q	T481M	G482C	Q484H	R620C	R623W	R623L	R638L	R638W
***k_cat_* (s^−1^)**	70.2 ± 3.6	53.9 ± 1.5	52.3 ± 1.6	26.5 ± 0.9	62.5 ± 1.0	45.1 ± 1.8	63.0 ± 1.6	41.1 ± 0.6	55.7 ± 1.8	15.6 ± 0.4	8.6 ± 0.3
***K_m_* (µM)**	12.7 ± 1.8	8.3 ± 0.8	10.4 ± 1.0	10.0 ± 1.1	8.8 ± 0.4	6.6 ± 1.0	11.2 ± 0.8	7.6 ± 0.4	9.8 ± 1. 0	3.5 ± 0.42	3.5 ± 0.5
***k_cat_/K_m_*** **(s^−1^/µM)**	5.5	6.5	5.0	2.6	7.1	6.8	5.6	5.4	5.7	4.5	2.5

**Table 2:** Binding affinity of DPP III variants for the Kelch domain compared to the affinity of the wild-type protein, expressed as the ratio *K_d_*(WT)/*K_d_*(variant). Measurements were performed in triplicate.

DPP III	*K_d_*(WT)/*K_d_*(mut) ^a^
WT	1.0
E451K	2.1
P479S	18.4
E480Q	0.1
T481M	0.1
G482C	0.8
Q484H	2.1
R510W	0.3
R623W	160.0 ^b^
R638L	2.0
R638W	2.0
R703C	1.7

^a^ The average error for the MST measurements is about 30%. ^b^ Matić et al., 2021 [17].

**Table 3:** Relative free energies of binding of two interactor proteins in the Kelch-DPP III complexes calculated by the MM-GBSA method. Values were calculated at intervals of 20 ns over MD trajectories, and the minimum value obtained for each of the independent trajectories is indicated. The full MM-GBSA energy profiles during the time of the trajectories can be found in Figure S2.

DPP III-Variant	Simulations	Time/ns	MM-GBSA-Min (kcal/mol)
WT	1	300	−51
	2	200	−80
	3	200	−73
	total	700	−80
P479S	1	300	−93
	2	200	−116
	3	200	−85
	total	700	−116
E480Q	1	300	−42
	2	200	−36
	3	200	−42
	total	700	−42
T481M	1	300	−63
	2	200	−52
	3	200	−63
	total	700	−63
G482C	1	300	−55
	2	200	−56
	3	200	−45
	total	700	−56
R510W	1	300	−69
	2	200	−64
	3	200	−49
	total	700	−69
R623W	1	300	−51
	2	200	−50
	3	200	−65
	total	700	−65

**Table 4:** Population of the intermolecular hydrogen bonds during the entire simulation time (700 ns) for each DPP III variant in the complex with the Kelch domain of KEAP1.

DPP III Variant	Total H-Bonds
WT	14.7
P479S	20.6
E480Q	8.8
T481M	13.1
G482C	10.4
R510W	13.6

## Supplementary Materials

The following supporting information can be downloaded at: https://www.mdpi.com/article/10.3390/ijms23041994/s1.
