# Supplementary material for: Interdisciplinary Study of the Effects of Dipeptidyl-Peptidase III Cancer Mutations on the KEAP1-NRF2 Signaling Pathway
Source: Int J Mol Sci. 2022 Feb 11;23(4):1994. doi: 10.3390/ijms23041994 (PMC8878202; doi:10.3390/ijms23041994)
Supplement: Supplementary file 1 [file ijms-23-01994-s001.zip › ijms-1560219-supplementary.pdf]

SUPPORTING INFORMATION

Interdisciplinary Study of the Effects of Dipeptidyl-peptidase III Cancer Mutations on the KEAP1-NRF2 Signaling Pathway

Sara Matić, Ana Tomašić Paić, Sandra Sobočanec, Marija Pinterić, Goran Pipalović, Monika Martinčić, Mihaela Matovina * and Sanja Tomić

Content

1. Table S1 2
2. Table S2 3
3. Table S3 4
4. Table S4 5
5. Figure S1 5
6. Figure S2 6
7. Figure S3 7

Table S1. The first a) and second b) sets of K_d values determined by initial fluorescence change measurements performed on the MST instrument for the binding of DPP III variants to a labeled Kelch domain. Measurements were performed in triplicate. For each set of measurements, the newly labeled Kelch was used.

a)

**DPP III**	**K_d (mol dm^{-3})**
**WT**	$(8,3 \pm 1,1)x10^{-7}$
**E451K**	$(4,0 \pm 0,5)x10^{-7}$
**P479S**	$(4,5 \pm 4,9)x10^{-8}$
**G482C**	$(1,0 \pm 0,4)x10^{-6}$
**Q484H**	$(3,9 \pm 1,1)x10^{-7}$
**R638L**	$(4,1 \pm 0,6)x10^{-7}$
**R638W**	$(4,1 \pm 0,5)x10^{-7}$
**R703C**	$(4,8 \pm 0,8)x10^{-7}$
**R703H**	$(1,0 \pm 0,1)x10^{-6}$

b)

**DPP III**	**K_d (mol dm^{-3})**
**DT**	$(1,9 \pm 0,7)x10^{-7}$
**E480Q**	$(1,6 \pm 0,4)x10^{-6}$
**T481M**	$(2,3 \pm 0,4)x10^{-6}$
**R510W**	$(6,3 \pm 2,0)x10^{-7}$
**V514M**	$(2,3 \pm 0,9)x10^{-7}$

Table S2. The intermolecular hydrogen bonds formed during MD simulations of the complex Kelch - DPP III WT and mutant variants in the intervals with minimum calculated MMGBSA energy.

DPP III	Kelch	WT	P479S	E480Q	T481M	G482C	R510W
Q463	P384			0.52			
E465	R336	0.35					
K466	Q337		0.54				
G467	N387		0.68				
G467	S383		0.43				
A468	Q337		0.50				
F469	Y334		0.81				
F469	R336						0.13
N470	Q337			0.37			
F471	R336			0.64			
Q473	Y572			0.27			
Q473	R336		0.13				
Q473	G574		0.42		0.41	0.24	0.65
Q473	H575	0.48	0.19				
Q473	Y572		0.21				
Q473	Q530					0.49	
E474	H575	0.55					
N478	R483				0.10		
P/S479	Q530	0.80	0.72	0.75	0.67	0.64	0.72
E/Q480	S555	0.86	0.96	0.94	1.00	0.97	0.98
E/Q480	R483	1.73	2.04	0.83	1.67	1.62	1.68
E/Q480	S508	0.98	0.93	0.49	0.90	0.92	1.00
E/Q480	R415	1.70			1.79	1.86	1.60
T/M481	S602		1.12			0.80	0.89
G/C482	Y334					0.18	
E483	N414	0.81					
E483	R415		1.87	0.90			
E483	S363		0.98				
E483	S602		0.95				
E483	N387			0.12	0.72	0.77	
E483	R380			0.93	0.87	0.94	0.85
Q484	R336				1.05		0.12
Q484	Y334			0.14			0.40
Q484	R380	0.82			0.56		
Q484	N387				0.88		
Q484	S363	0.12					
Q484	N381	0.59					
I485	N387	0.73					
Q486	Y334						0.16
Q486	R336						0.54
Q486	N387	1.02		0.47		0.73	0.14
Q486	D389	0.66					
Q486	R380		0.72	0.34			
W489	D385			0.37			
R490	P384		0.64				
R490	N387		0.92				
R490	S383		0.39				
K498	D385			0.70			
E581	R459		0.74		1.74		1.38
E619	T481	0.61	0.47				
R620	D479	1.32	1.27				
R620	T481				0.20		0.23
R623	G480	0.27					
R623	C434	0.30					
R624	C434					0.29	
R624	H436				0.51		
R624	D479		0.51				
D633	R380		1.41				

D633	N387						1.53
A635	D385						0.25
A635	T388						0.36

Table S3. Primers used for site directed mutagenesis in DPP III genes by QuickChange mutagenesis protocol. For each of the introduced mutations, a primer is listed in the main (F, forward) and complementary (R, reverse) chain, in the 5'→3 'direction.

mutation of DPP III	-primer sequence-
E451K	F- 5'-CAGGTGGGCCTGCACAAGCTGCTGGGCCATGGC- 3'
	R- 5'-GCCATGGCCCAGCAGCTTGTGCAGGCCCACCTG- 3'
P479S	F- 5'-GAAACAGTGATCAACTCAGAGACGGGCGAGC-3'
	R- 5'-GCTCGCCCGTCTCTGAGTTGATCACTGTTTC- 3'
E480Q	F- 5'-GTGATCAACCCACAGACGGGCGAGCAG- 3'
	R- 5'-CTGCTCGCCCGTCTGTGGGTTGATCAC- 3'
T481M	F- 5'-GATCAACCCAGAGATGGGCGAGCAGATTC- 3'
	R- 5'-GAATCTGCTCGCCCATCTCTGGGTTGATC- 3'
G482C	F- 5'-CAACCCAGAGACGTGCGAGCAGATTCAG-3'
	R- 5'-CTGAATCTGCTCGCACGTCTCTGGGTTG-3'
Q484H	F- 5'-GAGACGGGCGAGCACATTCAGAGCTGGTATC-3'
	R- 5'-GATACCAGCTCTGAATGTGCTCGCCCGTCTC-3'
R638L	F- 5'-GATGTGGCCGGAGGGCTGGCCCTGTACGAGG-3'
	R- 5'-CCTCGTACAGGGCCAGCCCTCCGGCCACATC-3'
R638W	F- 5'-GATGTGGCCGGAGGGTGGGCCCTGTACGAGG-3'
	R- 5'-CCTCGTACAGGGCCCACCCTCCGGCCACATC-3'
R703C	F- 5'-CCATCCTCTGGGAAACACTCAGAGAAGGATCGG -3'
	R- 5'-CCGATCCTTCTCTGAGTGTTTCCCAGAGGATGG -3'
R703H	F- 5'-GTCCATCCTCTGGGAAATGCTCAGAGAAGGATCGG -3'
	R- 5'-CCGATCCTTCTCTGAGCATTTCCCAGAGGATGGAC -3'

Table S4. Primers used for RT-qPCR

Gene/Primers	Primer sequence	Product size/pb	Reaction Efficiency /%	Ref.
**GAPDH**<br>GAPDH-FOR/REV	GAGTCAACGGATTTGGTCGT<br>GACAAGCTTCCCGTTCTCAG	185	96,31	(Wang et al., 2017)
**TUBG1**<br>TUBG1-QP-F/R	TCAGGGACCTCACGCATCTCTTTC<br>TGTGTTTGCAGGCCAACAGAGAG	60	102,53	QuantPrime*
**NRF2**<br>NRF2-Ori-F/R	CACATCCAGTCAGAAACCAGTGG<br>GGAATGTCTGCGCCAAAAGCTG	112	96,48	Origene primers
**NQO1**<br>NQO1-Ori-F/R	CCTGCCATTCTGAAAGGCTGGT<br>GTGGTGATGGAAAGCACTGCCT	119	89,02	Origene primers
**HMOX1**<br>HMOX1-Ori-F/R	CCAGGCAGAGAATGCTGAGTTC<br>AAGACTGGGCTCTCCTTGTTGC	144	98,97	Origene primers
**PRDX1**<br>PRDX1-F/R	GGGCACACAAAGGTGAAGTC<br>GCTGTTATGCCAGATGGTCAG	104	96,07	(Bollong et al., 2018)
**SOD1**<br>SOD1-QP-F/R	GCGGAGGTCTGGCCTATAAAGTAG<br>AGGAGACTACGACGCAAACCAG	62	105,47	QuantPrime*
**GCLM**<br>GCLM-QP-F/R	TCAGTCCTTGGAGTTGCACAGC<br>TCCTCCCAGTAAGGCTGTAAATGC	104	100,55	QuantPrime*
**SLC7A11 (xCT)**<br>SLC7A11-Ori-F/R	TCCTGCTTTGGCTCCATGAACG<br>AGAGGAGTGTGCTTGCGGACAT	122	96,83	Origene primers
**GPX1**<br>GPX1-Ori-F/R	GTGCTCGGCTTCCCGTGCAAC<br>CTCGAAGAGCATGAAGTTGGGC<br>AGGAGACTACGACGCAAACCAG<br>CTCGAAGAGCATGAAGTTGGGC	124	104,26	Origene primers

* primers constructed with QuantPrime software

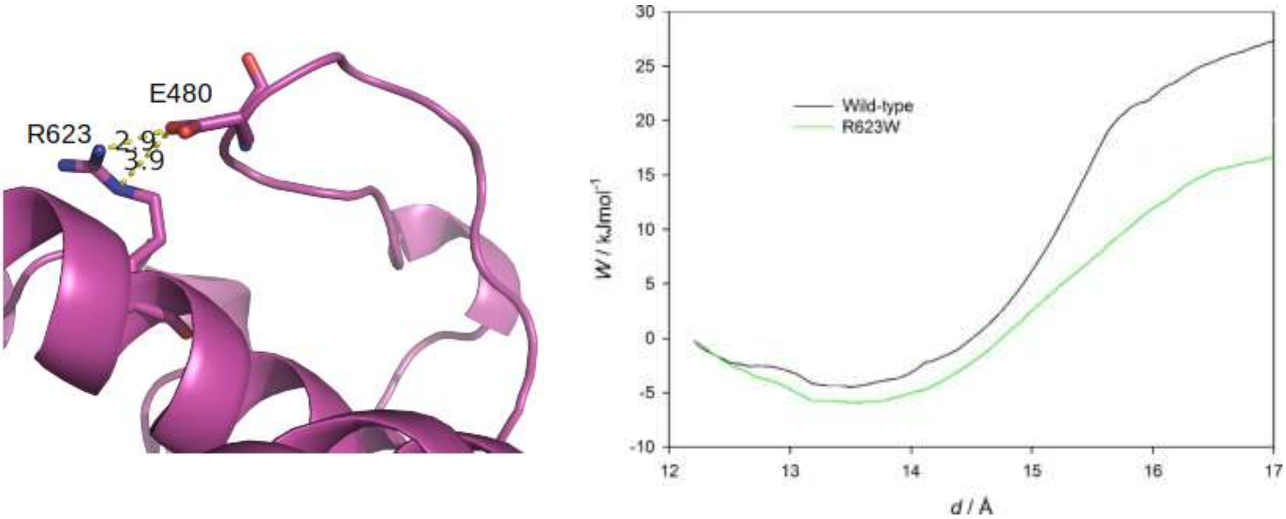

Figure S1. Left – Interaction between E480 and R623. Right – Work needed to detach the $E^{480}TGE^{483}$ loop from the surface of the wild-type DPP III and the R623W variant.

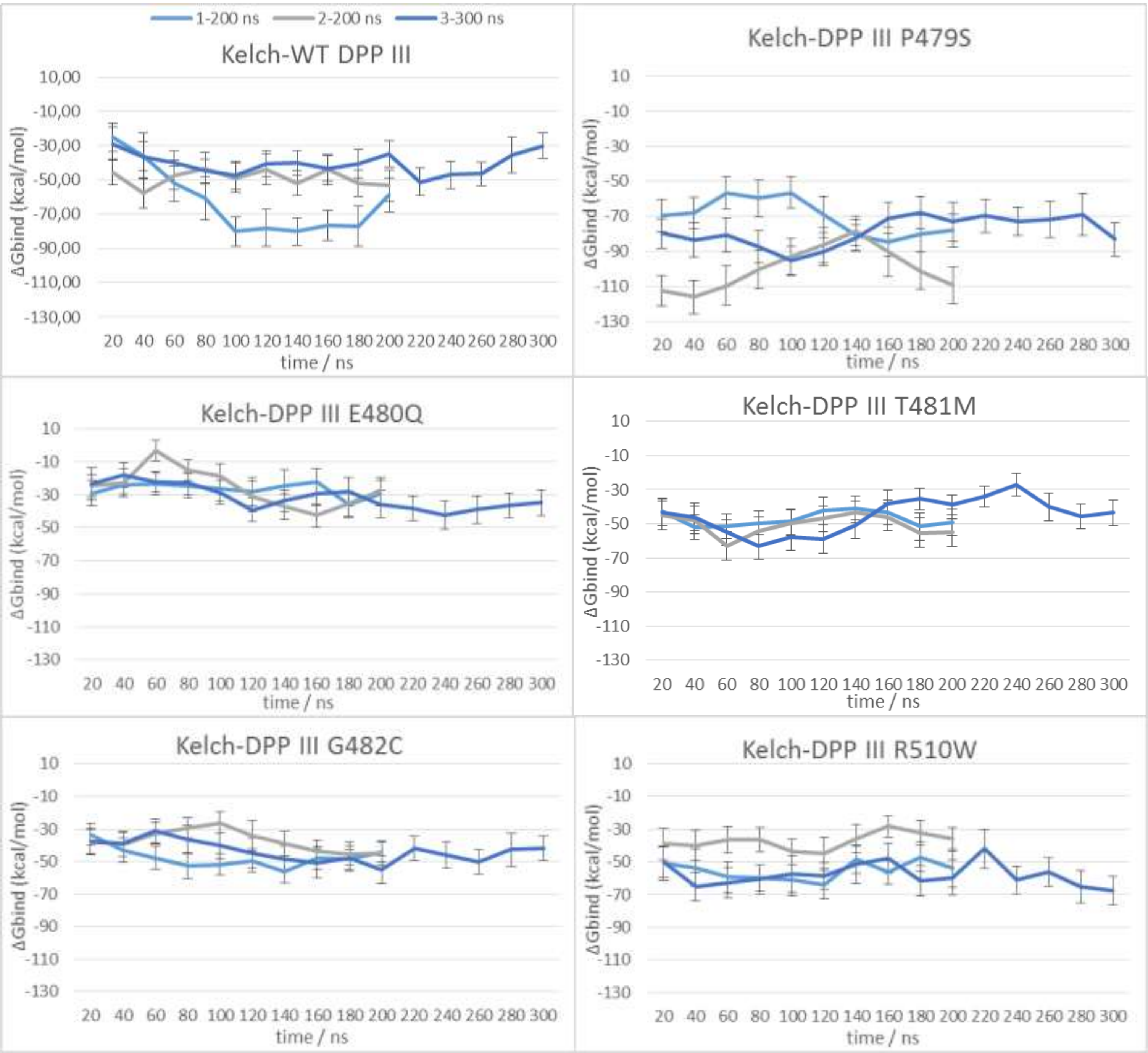

Figure S2. Relative free energies of binding of two interactor proteins in the Kelch - DPP III complexes calculated by the MM-GBSA method. Values were calculated at intervals of 20 ns for each of the three independent MD trajectories for DPP III variants in the complex with Kelch domain, and standard deviations are given as error bars.

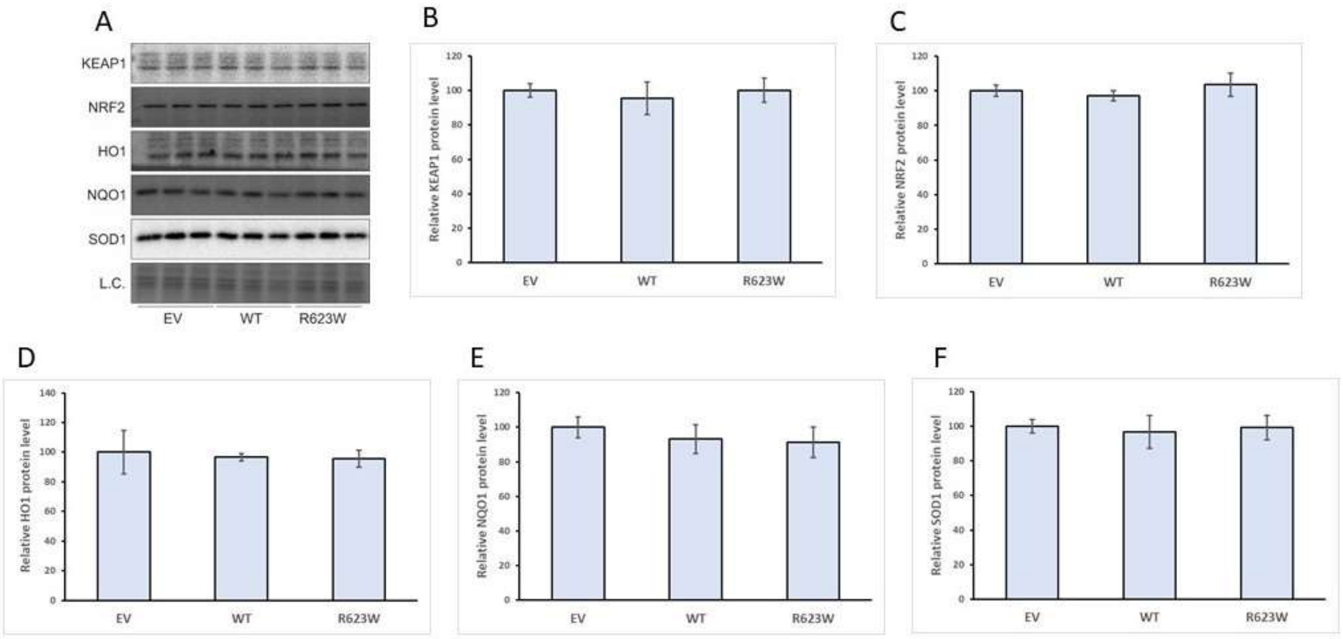

Figure S3. Western blot analysis of the expression of KEAP1, NRF2, HO1, NQO1 and SOD1 in EV, WT DPP III and DPPIII-R623W transfected HEK293T cells (A); A graphical display of the averaged densitometry values obtained by the analysis of the western blot bends in ImageLab v. 6.1.0. (Bio-Rad Laboratories, Inc.), normalized with loading control (L.C.) for KEAP1 (B), NRF2 (C), HO1 (E), NQO1 (D) and SOD1 (F) protein expression is presented.
